# key-fg DETR based camouflaged locust objects in complex fields

**DOI:** 10.3389/fpls.2025.1565739

**Published:** 2025-07-23

**Authors:** Dongmei Chen, Peipei Cao, Zhihua Diao, Yingying Dong, Jingcheng Zhang

**Affiliations:** ^1^ College of Artificial Intelligence, Hangzhou Dianzi University, Hangzhou, China; ^2^ School of Electrical Information Engineering, Zhengzhou University of Light Industry, Zhengzhou, China; ^3^ Key Laboratory of Digital Earth Science, Aerospace Information Research Institute, Chinese Academy of Sciences, Beijing, China

**Keywords:** pest recognition, camouflaged target, object detection, crop protection, transformer networks

## Abstract

**Introduction:**

In real agricultural environments, many pests camouflage themselves against complex backgrounds, significantly increasing detection difficulty. This study addresses the challenge of camouflaged pest detection.

**Methods:**

We propose a Transformer-based detection framework that integrates three key modules: 1.Fine-Grained Score Predictor (FGSP) – guides object queries to potential foreground regions; 2.MaskMLP generates instance-aware pixel-level masks; 3.Denoising Module and DropKey strategy – enhance training stability and attention robustness.

**Results:**

Evaluated on the COD10k and Locust datasets, our model achieves AP scores of 36.31 and 75.07, respectively, outperforming Deformable DETR by 2.3% and 3.1%. On the Locust dataset, Recall and F1-score improve by 6.15% and 6.52%, respectively. Ablation studies confirm the contribution of each module.

**Discussion:**

These results demonstrate that our method significantly improves detection of camouflaged pests in complex field environments. It offers a robust solution for agricultural pest monitoring and crop protection applications.

## 1 Introduction

**Figure 1:**
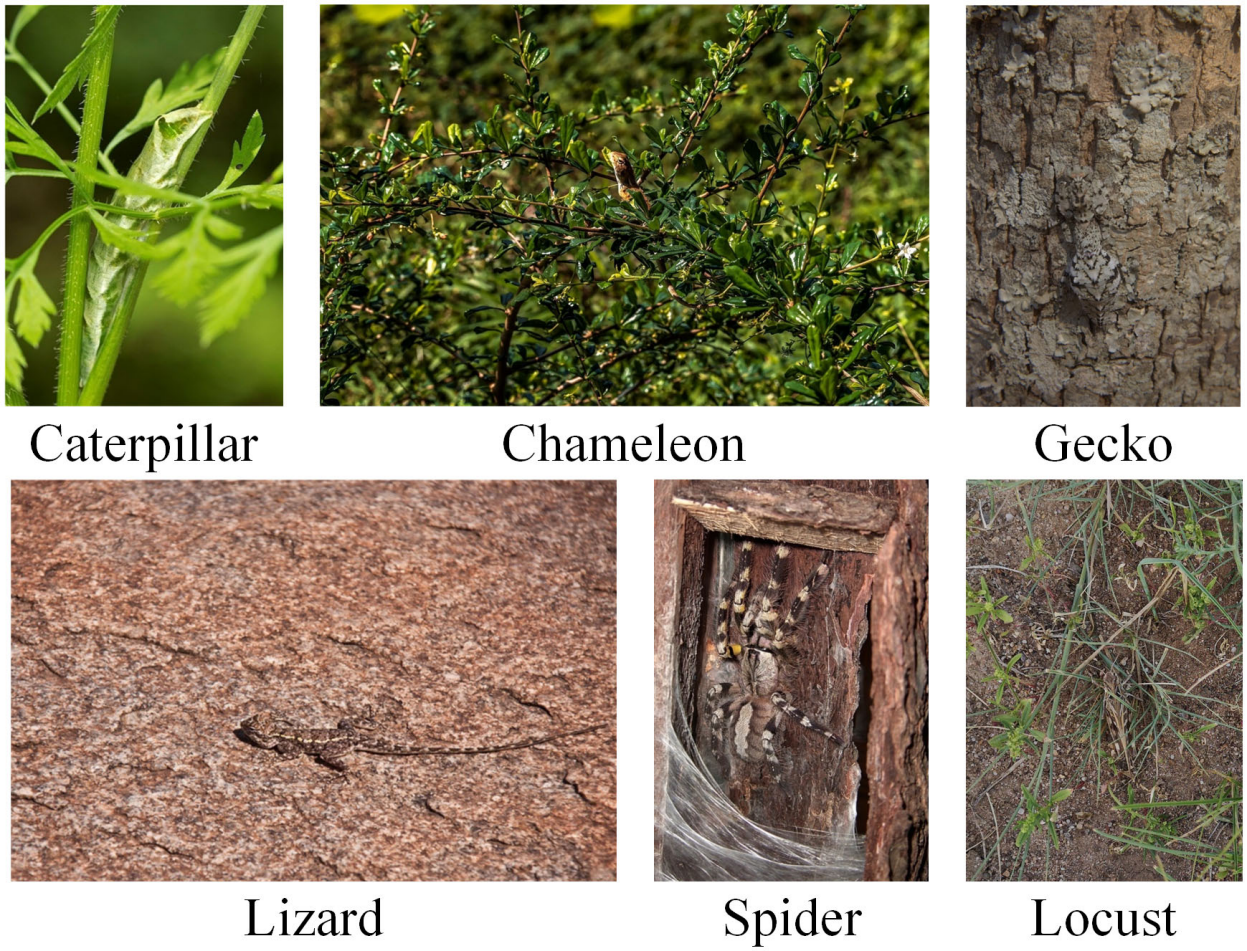
Examples of camouflaged pest insects across different categories.

Insects have evolved remarkable camouflage capabilities over long evolutionary processes to evade predators and adapt to their environments. This phenomenon, commonly referred to as mimicry, involves an organism resembling another organism or its surroundings to gain a survival advantage (Cédric et al., 2019; Stevens et al., 2009), and plays a crucial role in insect survival. Species such as the leaf butterfly exhibit wing patterns that closely resemble dried leaves, while stick insects mimic tree branches with striking precision, rendering them virtually indistinguishable from their environments (Endler, 1981). These adaptations not only assist insects in predator avoidance but also render them nearly “invisible” to human observers, as illustrated in **Figure 1** (Troscianko et al., 2016). While some camouflaged insects, such as chameleons, pose minimal threats to human activities and require limited monitoring, others—such as locusts—have profound implications for agriculture, ecosystems, and public health, necessitating timely and accurate detection (Chudzik et al., 2020). Locusts, in particular, are notorious for their prolific reproduction and migratory behaviors, often resulting in devastating crop losses (Adriaansen et al., 2023). Similarly, other camouflaged insects, such as mosquitoes, serve as critical vectors for disease transmission while simultaneously avoiding detection due to their natural concealment abilities (Foster and Walker, 2019). These challenges underscore the urgency of developing robust pest detection systems capable of identifying camouflaged targets within complex natural environments. Therefore, there is an urgent need for detection frameworks that can accurately distinguish camouflaged pests from intricate backgrounds while maintaining high localization precision and robustness against environmental noise (Li et al., 2021b).

Early approaches to insect identification primarily relied on manual observation and traditional image processing techniques, leveraging features such as color, texture, and morphology (Weeks et al., 1999; Gaston and O'Neill, 2004; Li and Xiong, 2018). However, these methods often perform poorly in real-world scenarios where environmental complexity significantly hinders effective feature extraction, particularly for camouflaged insects (Li et al., 2021a). With the advent of smart agriculture, the integration of Internet of Things (IoT) and artificial intelligence (AI) technologies has provided promising avenues for real-time data acquisition and intelligent decision-making. Recent studies (Khan et al., 2022a, 2022; Bashir et al., 2023) have demonstrated the potential of IoT-assisted systems in applications such as soil fertility mapping, context-aware evapotranspiration estimation, and optimization of reference evapotranspiration, highlighting the transformative role of AI and IoT in agricultural management. These advancements further accentuate the need for intelligent pest detection systems to complete the digital agriculture pipeline. In recent years, deep learning-based models have significantly improved insect detection accuracy by learning discriminative features directly from data (Preti et al., 2021). For instance, Wang et al. (2020) applied YOLOv3 (Redmon and Farhadi, 2018) to detect 24 pest species, Liu et al. (2023) enhanced pest recognition using GA-Mask R-CNN, and Teng et al. (2022) proposed MSR-RCNN with super-resolution and feature-weighting components to address the detection of visually similar pests. Bai et al. (2022) further demonstrated (Bochkovskiy et al., 2020) that integrating the MOG2 algorithm with YOLOv4 can enhance locust detection performance in video sequences. Qi et al. (2022) trained the Pest24 dataset with an improved Deformable DETR, achieving significant results. Despite these advances, camouflaged pest detection remains particularly challenging due to the subtle visual distinctions between pests and their backgrounds, compounded by environmental noise.

Camouflaged insect detection is closely related to the broader field of camouflaged object detection (COD) in computer vision, which aims to identify objects that seamlessly blend into their surroundings (Lv et al., 2021). Although COD has achieved notable success in domains such as medical imaging and military surveillance, many existing models primarily focus on segmentation quality rather than object-level detection and classification, thus limiting their applicability to agricultural contexts (Cheng et al., 2017). Recent developments in Transformer-based architectures have demonstrated promising potential in COD tasks. Mao et al. (2021) introduced T2Net with Swin Transformer to capture global contextual features, while Yang et al. (2021) proposed UGTR to enhance the focus on uncertain regions by combining CNNs and Transformers. Nonetheless, these models often suffer from sensitivity to noise and difficulty in capturing fine-grained local details. Huang et al. (2023) further introduced FSPNet, a hierarchical Transformer-based architecture with enhanced locality modeling and progressive feature aggregation, achieving state-of-the-art performance across multiple COD datasets. Recent work (Khan et al., 2024, 2023) has further demonstrated the versatility of Transformer-based frameworks in handling complex and noisy data across diverse domains, indicating their strong adaptability. Inspired by these advances, this study leverages Transformer-based detection to better address the challenges of camouflaged pest identification. Unlike tasks in the medical or military domains, agricultural pest detection demands not only accurate localization but also species-level classification, necessitating models that are both precise and efficient (Cheng et al., 2017). To bridge this gap, we propose an efficient end-to-end model, Transformer-based detection method for camouflaged objects (key-fg DETR), specifically designed for the detection and recognition of camouflaged agricultural pests. Locust detection poses particular challenges due to their green coloration, which closely blends with surrounding vegetation, making visual differentiation difficult. Additionally, their flat body structure allows them to adhere tightly to plant surfaces, further enhancing their concealment. Moreover, their migratory and swarm behaviors often lead to sudden, large-scale outbreaks that are difficult to monitor and control. Given the extensive agricultural damage caused by locust infestations, the ability to detect them at an early stage is especially critical.

The proposed method enhances detection performance by integrating global and local feature information. Specifically, a multi-scale feature extraction strategy captures discriminative pest features across varying levels of abstraction. A Fine-Grained Score Predictor (FGSP) module refines local feature selection, while pixel-level instance masks generated by the MaskMLP module improve the localization of occluded targets. Additionally, a denoising module is incorporated to suppress background interference and stabilize the matching process. Extensive experiments conducted on the COD10k dataset and a custom locust dataset validate the effectiveness and generalizability of the proposed approach under complex background conditions. The major contributions of this work can be summarized as follows:

1. We design a multi-scale feature extraction framework that enhances pest identification across different scales and cluttered backgrounds, addressing the issue of insufficient feature representation for small and camouflaged targets.
2. We propose the Fine-Grained Score Predictor (FGSP) module to selectively focus on informative local features, thereby improving the discriminability of camouflaged pests with subtle appearance variations.
3. We develop the MaskMLP module to generate pixel-level instance masks, significantly boosting localization accuracy especially under conditions of partial occlusion.
4. We introduce a denoising module that effectively suppresses environmental noise and stabilizes the matching between predictions and targets during training, enhancing robustness.
5. Through extensive experiments on the COD10k and locust datasets, we validate the effectiveness of our approach via comparative analysis, ablation studies, and detailed visual evaluations.

The remainder of this paper is organized as follows. Section 2 details the methodological innovations, including the construction of the locust dataset with camouflage degree quantification, experimental environment setup, and data augmentation strategies, with a particular focus on the key-fg DETR framework and its four core components: MaskMLP, the denoising module, DropKey for enhancing attention reliability, and FGSP for fine-grained feature extraction. Section 3 presents the experimental results based on comparative studies, ablation experiments, and visualization analysis on the COD10k and locust datasets. Section 4 concludes the paper and discusses potential future research directions for intelligent agricultural detection.

## 2 Materials and methods

### 2.1 Dataset

Locusts are one of the most destructive migratory pests to crops. They exhibit great diversity in species and morphology, inhabit complex environments, and are adept at camouflage, making them challenging for detection models and prone to recognition errors (Ye et al., 2014). The locust dataset used in this study was sourced from the publicly available GHCID dataset (Chudzik et al., 2020), containing 2,379 images. Annotation was performed through the EasyDL platform, generating 2,789 annotation files in COCO format. Following the method proposed by Fan et al. (2020), the input images were analyzed. However, it should be noted that the camouflage degree evaluation method proposed by Fan et al. was primarily designed for segmentation tasks, focusing on pixel-level camouflage characteristics, and there is currently no dedicated evaluation method for camouflage degree specifically for object detection tasks. Therefore, this chapter proposes a new camouflage degree (CD) evaluation metric tailored for object detection, as shown in Equation 1, to determine whether a dataset belongs to the camouflaged object category. The computation is based on the values derived from Equation 2, following these steps: First, the RGB histograms of the foreground and background regions are calculated for each color channel. Then, the histograms for each channel are normalized by converting pixel counts into probability values.


(1) $$\text{CD}=\frac{T_{gc}<0.9}{T_{gc}\geq 0.9}$$

(2) $$T_{gc}=\sum_{i=1}^{n}\frac{{\left(h_{1}\left(i\right)-h_{2}\left(i\right)\right)}^{2}}{h_{1}\left(i\right)+h_{2}\left(i\right)}$$

in which, ${\text{T}}_{gc}$ represents the $\chi^{2}$ distances between the foreground and background regions in the image. We calculated the RGB histogram of each color channel for both the foreground and background regions. Subsequently, we normalized the histogram of each channel, converting pixel counts into probability values, in which ${\text{h}}_{1}\left(\text{i}\right)$ and ${\text{h}}_{2}\left(\text{i}\right)$ respectively represent the probability values of the i -th interval in the foreground and background histograms. The $\chi^{2}$ distances of each channel are added together to obtain the total $\chi^{2}$ distance. The smaller the $T_{gc}$ distance, the more similar the two histograms are; the larger it is, the greater the difference.

**Figure 2:**
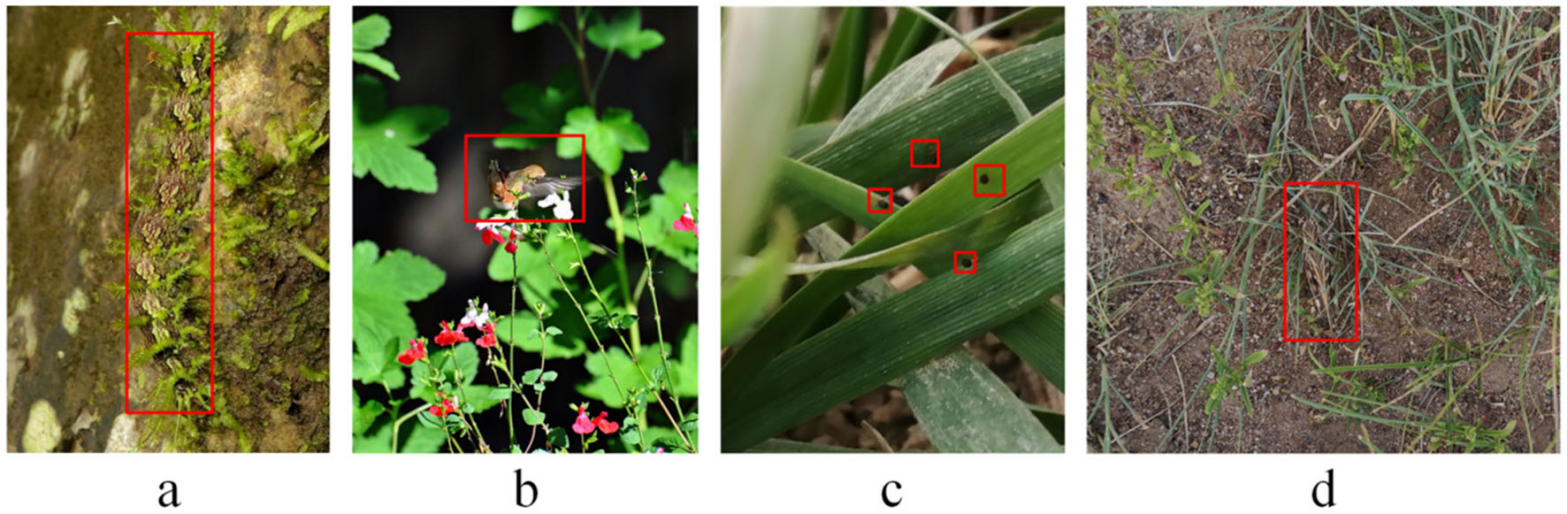
**(a)** COD10k dataset, **(b)** COCO2017 dataset, **(c)** MTC-PAWPD dataset and **(d)** Locust dataset.

To further validate that the locust dataset belongs to the camouflaged object category, we also collected 3,298 images and 3,330 instances from the COD10k dataset, encompassing 51 biological categories such as ants, bugs, cats, caterpillars, centipedes, and chameleons. The images of the specific datasets are shown in **Figure 2**. All images were re-annotated using the EasyDL platform. The camouflage degree (CD) for each object was calculated based on Equation 2, and the final ratio was obtained using Equation 1, resulting in a CD value of 1.03. Additionally, a comparative analysis was performed on the COCO2017 dataset, which contains 80 categories, 5,000 images, and 36,781 objects (Lin et al., 2014). The computed CD value for COCO2017 was 0.26, indicating that it is not a typical camouflage dataset. Furthermore, the MTC-PAWPD dataset, which includes images of pests such as planthoppers, aphids, and wheat spiders collected in complex natural field environments (containing 19,970 instances), was also analyzed. The resulting CD value was 0.10, again confirming that MTC-PAWPD is not a typical camouflaged dataset (Chen et al., 2024). **Table 1** presents the statistics of the locust dataset, including 2,379 images and 2,789 objects. Based on the above calculations, the final CD value for the locust dataset was determined to be 1.05. The CD value of the locust dataset is not only significantly higher than that of the non-camouflaged datasets COCO2017 and MTC-PAWPD, but also higher than the classical camouflage dataset COD10k. This result demonstrates that the locust dataset is a typical camouflaged object dataset, making it suitable for camouflage object detection tasks.

**Table 1:** Comparison of different datasets.

Dataset	$T_{gc}$ <0.9	$T_{gc}\geq$ 0.9	*CD*	Number of objects
COD10k	1689	1641	1.03	3330
COCO2017	7687	29094	0.26	36781
MTC-PAWPD	1746	18224	0.10	19970
Locust	1426	1363	1.05	2789

### 2.2 Implementation details

The computer configuration used for processing the locust images comprised an Intel(R) Xeon(R) Platinum 8352V CPU @ 2.10GHz, 64GB of RAM, an NVIDIA GeForce RTX 4090 GPU with 24GB of memory, and the Linux Ubuntu 22.04 operating system. Due to the limited size of the dataset, the hold-out method (Raschka, 2018) was adopted to split the data into training and validation sets in a 7:3 ratio, in order to avoid overfitting caused by insufficient sample size. This strategy helps maximize the use of available data for both training and validation, thereby enhancing the robustness and reliability of the performance evaluation. As illustrated in **Table 2**, During the model training phase, we used the Adam optimizer with an initial learning rate of 0.001 and a weight decay factor of 0.0005 to prevent overfitting. The learning rate was adjusted using a cosine annealing schedule. The batch size was set to 4, and the total number of training epochs was 50. In addition, we conducted sensitivity analyses on key hyperparameters (learning rate and batch size) to assess their impact on model performance. For the learning rate, we tested values of 0.0001, 0.001, and 0.01. The results showed that 0.001 achieved the best balance between convergence speed and accuracy. For batch size, we tested 16, 8, and 4. Due to the large size of the model, larger batch sizes could not be accommodated within GPU memory. Ultimately, a batch size of 4 was found to offer the best trade-off between memory usage and training stability.

**Table 2:** Hyperparameter settings for model training.

Hyperparameter	Value
Learning Rate	0.001
Optimizer	Adam
Weight Decay	0.0005
Learning Rate Decay	Cosine Annealing
Batch Size	4

**Figure 3:**
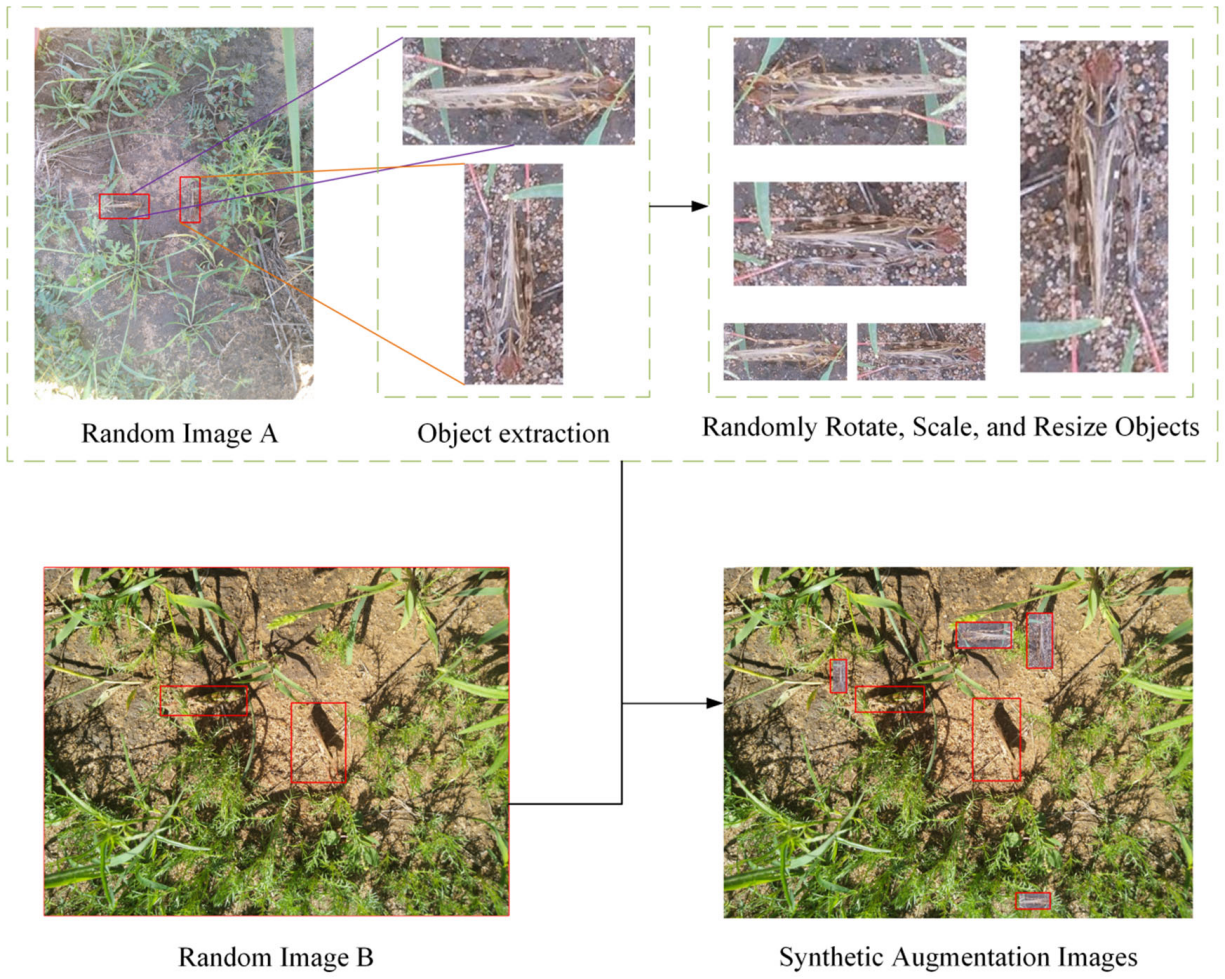
Data enhancement procedure.

To improve the model’s robustness and performance, data augmentation techniques have been widely applied in the training process (Rebuffi et al., 2021). In the study, we implemented several data augmentation techniques such as paste-copy, zoom in/out, rotation, etc., on different images to augment the dataset and improve the robustness of the model. The data augmentation results are illustrated in **Figure 3**. First, we randomly selected an object from an image and performed random rotations, scaling, or other operations on it. Then, we randomly paste these objects onto another image to generate augmented images. Adding these augmented images to the dataset, the total dataset contains 5522 images. By increasing the diversity of the dataset, we reduced the risk of overfitting, allowing the model to better adapt to various complex real-world scenarios.

### 2.3 key-fg DETR model architecture

**Figure 4:**
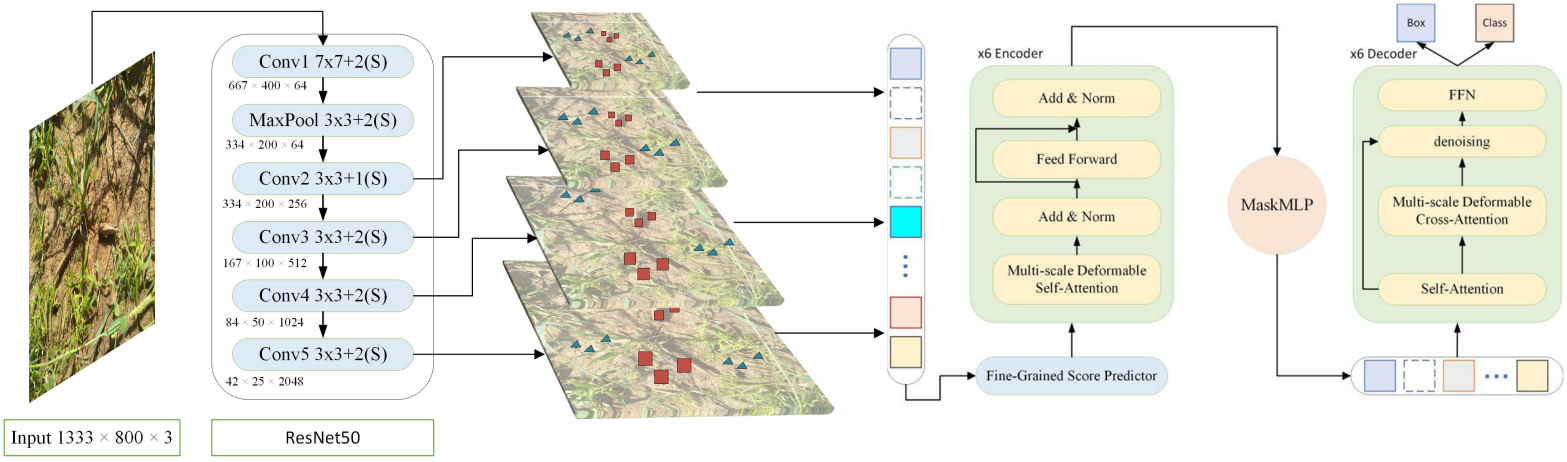
Key-fg DETR model architecture.

Methods based on convolutional neural networks (CNNs) have certain limitations in object detection tasks, such as difficulty in capturing global contextual information and a heavy reliance on predefined anchor points (Luo et al., 2016). CNNs rely heavily on local receptive fields and exhibit strong inductive bias, which makes them effective in many visual tasks but less flexible when dealing with high background similarity, occlusion, and long-range dependencies challenges commonly encountered in camouflaged pest detection. In contrast, Transformer-based detection methods overcome these limitations by capturing long-range dependencies (Vaswani et al., 2017), demonstrating stronger adaptability in complex scenarios. Compared to convolutional neural networks (CNNs), Transformer architectures demonstrate significant advantages in camouflaged object detection tasks. First, Transformers can effectively model global and long-range dependencies across spatial positions, which is crucial for distinguishing subtle differences between camouflaged pests and complex backgrounds. Second, Transformers support full parallelism during training, greatly improving computational efficiency. In addition, Transformers have a lower inductive bias, enabling more flexible and task-specific feature representation learning, which enhances the extraction features and further improves detection accuracy and model adaptability. This advantage is especially evident in agricultural environments with high occlusion and background similarity. Based on this, this study proposes an innovative solution-the key-fg DETR framework. The approach focuses on enhancing the model’s ability to capture fine-grained details and robustly handle multi-scale features. By integrating advanced attention mechanisms and refined prediction strategies, the proposed method effectively improves detection performance and robustness. As illustrated in **Figure 4**, the key-fg DETR model first feeds the input image (resized to 1333×800) into a ResNet-50 backbone to extract multi-level semantic features (He et al., 2016). Specifically, the model first applies a 7×7 convolution kernel with a stride of 2, producing a feature map of size 667×400×64. This is followed by a 3×3 max pooling layer with a stride of 2, yielding a feature map of 334×200×64. Subsequently, the following convolutional stages are applied: Conv2_x (3×3 convolution, stride 1, output size: 334×200×256), Conv3_x (3×3 convolution, stride 2, output size: 167×100×512), Conv4_x (3×3 convolution, stride 2, output size: 84×50×1024), and Conv5_x (3×3 convolution, stride 2, output size: 42×25×2048). All convolutional layers in the backbone utilize Batch Normalization and ReLU activation functions. These stages progressively extract multi-scale feature maps, gradually reducing spatial resolution while increasing semantic abstraction. The resulting features are then fed into the Fine-Grained Score Prediction (FGSP) module, which emphasizes critical local regions, thereby improving the representational precision of the encoder-particularly beneficial for detecting objects with high background similarity. The encoder employs a multi-scale deformable self-attention mechanism to model long-range dependencies and complex contextual interactions, enhancing the model’s ability to distinguish camouflaged objects from the background.

To address occlusion between targets, a dual-branch MaskMLP module is introduced in the decoder. This module consists of a spatial branch and a channel branch that work collaboratively to generate pixel-level masks, effectively suppressing boundary noise and irrelevant channel information. The output of this module is passed through a Sigmoid activation function to produce soft masks with values in the range [0, 1], enabling fine-grained spatial refinement. In addition, a Denoising Branch is incorporated in the decoding phase to stabilize the matching process between object queries and ground truth labels. During training, noisy queries are injected and trained in parallel with the main path, which improves the model’s robustness and convergence speed, especially under challenging conditions such as complex backgrounds and heavily occluded camouflaged objects.

#### 2.3.1 MaskMlp

To address the challenges posed by the diverse poses, complex shapes, and mutual occlusions of locusts in images, we introduced the MaskMLP module into our model. This module enhances the model’s ability to represent target regions through a dual-branch collaboration mechanism, significantly improving detection accuracy in occlusion scenarios.

**Figure 5:**
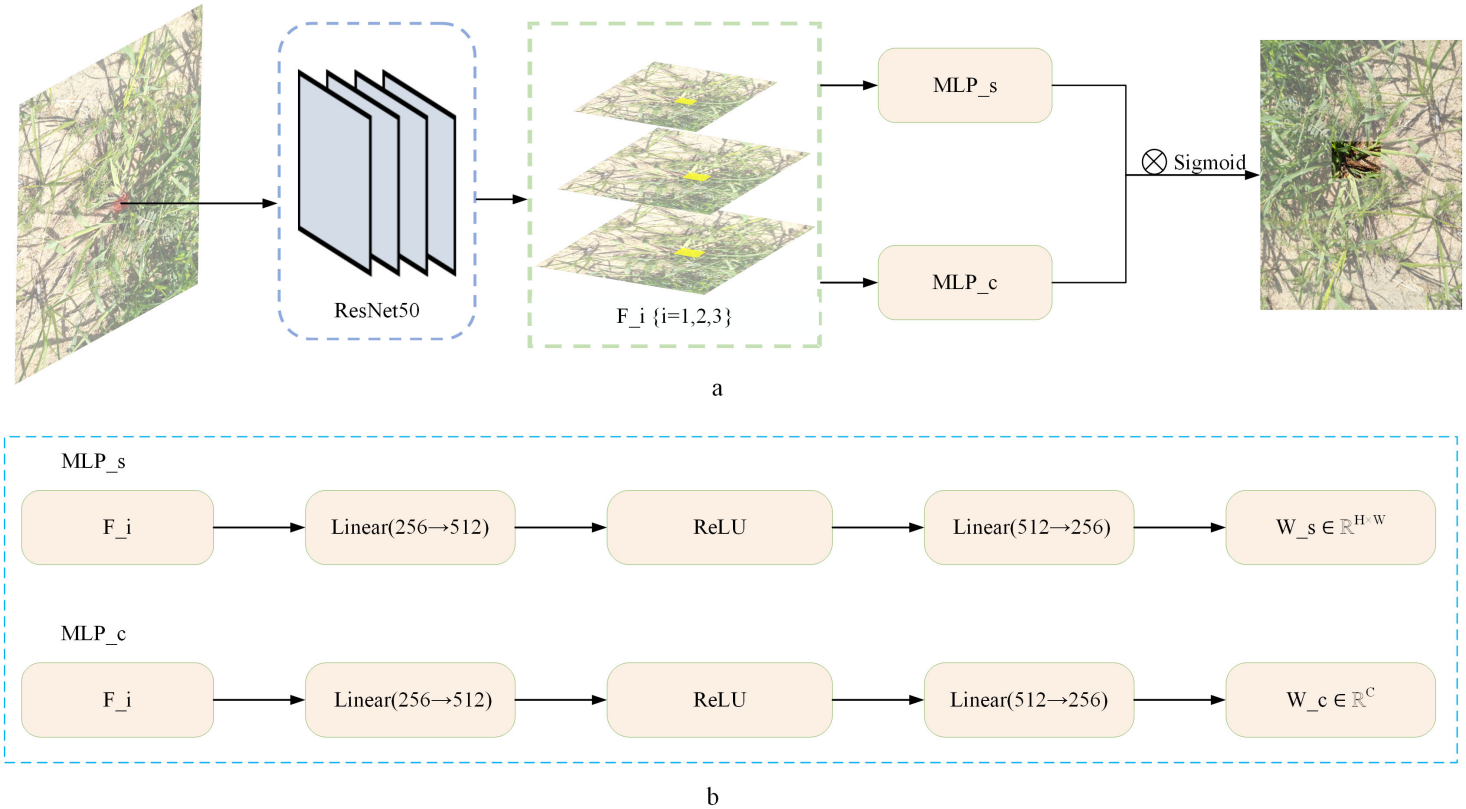
**(a)** Application of MaskMLP in occluded locust detection. **(b)** Structure of the dual-branch MaskMLP module. Multi-scale features F_i {i=1,2,3} from ResNet50 are processed by spatial branch MLP_s and channel branch MLP_c to generate spatial weights W_s and channel weights W_c.

As shown in **Figure 5**, the input image is first processed by ResNet50 to extract multi-scale feature maps, which are then simultaneously fed into two parallel branches. The spatial branch assigns lower weights to the features at occluded boundaries to suppress interference, generating a spatial weight map (height×width) that reflects the weight distribution at different locations. Meanwhile, the channel branch deactivates channels related to occluded regions, generating a channel weight vector to enhance feature selectivity. The outputs of both branches are fused via an outer product operation and activated by a Sigmoid function to generate a mask map, which guides the model to focus more precisely on the non-occluded regions. This mechanism enables the model to distinguish between background and foreground areas, even when they have similar appearances, and significantly improves the localization accuracy of occluded locusts. Experimental results demonstrate that the MaskMLP module improves AP_50_ by 15.6% under occlusion scenarios (see Section 3.2).

#### 2.3.2 Denoising model in decoder

To improve the model’s detection accuracy and performance, we combined the attention module of the original Transformer Encoder with deformable convolutions (Dai et al., 2017). This allows the module to focus only on sampling key points around the reference point, thereby addressing the slow convergence issue of DETR. The computation of the multi-scale deformable attention module is in Equation 3:


(3) $$\begin{array}{c} \text{MSDeformAttn}\left(z_{q},{\hat{p}}_{q},{\left\{{\text{t}}^{l}\right\}}_{l=1}^{L}\right)\\ =\sum_{m=1}^{M}W_{m}\left[\sum_{l=1}^{L}\sum_{k=1}^{K}A_{mlqk}\cdot W_{m}^{'}{\text{t}}^{l}\left(x_{l}\left({\hat{p}}_{q}\right)+\text{Δ}p_{mlqk}\right)\right] \end{array}$$

where m represents the index of the attention head, l represents the index of the feature map level, and k represents the index of the sampled point. $W_{m}^{'}$ ∈ $R^{\frac{C}{M}\times C}$ and $W_{m}$ ∈ $R^{C\times \frac{C}{M}}$ are learnable weights, while $\text{Δ}p_{mlqk}$ and $A_{mlqk}$ denote the sampling offsets and attention weights of the k -th sampled point in the m -th attention head respectively. $x_{l}\left({\hat{p}}_{q}\right)$ scales ${\hat{p}}_{q}$ back to each layer of the input feature map.

Due to the introduction of deformable attention mechanism in Deformable DETR, the model is allowed to learn non-rigid deformations of objects. However, learning deformations can become challenging due to the instability of the matching process. Using Denoising can improve the learning stability of deformation objects through denoising tasks, reducing the difficulty of offset learning caused by the instability of the matching process (Li et al., 2022). Moreover, the dynamic nature of the matching process in Deformable DETR may lead to inconsistencies in the predicted boxes matched to GT boxes for each query, which can have a negative impact on learning locust objects. The denoising task introduced by DN may help alleviate the instability of the matching process, improving the consistency between predicted boxes and ground truth boxes. Additionally, denoising task can serve as an additional learning task to learn the offsets relative to anchors in a more direct manner, which can accelerate the convergence speed of the key-fg DETR model.

The decoder contains both cross-attention and self-attention mechanisms, utilizing object queries as key elements. In cross-attention, object queries retrieve features from the encoder’s output feature map. Conversely, in self-attention, object queries interact amongst themselves. Given that the deformable attention module is tailored for handling convolutional feature maps, we substitute only the cross-attention modules with multi-scale deformable attention modules, while leaving the self-attention modules intact.

#### 2.3.3 Enhancing attention stability with DropKey

In the self-attention mechanism, DropKey introduces randomness into the attention matrix more selectively, thus acting as a regularization technique (Li et al., 2023). This helps reduce the risk of the model overly relying on specific patterns. Deformation objects often involve complex relationships and local features, and introducing DropKey can improve the learning stability of these relationships. Additionally, in certain cases, global dropout can lead to the loss of some global correlation information during learning (Srivastava et al., 2014). The introduction of DropKey allows for more precise control over which positions of attention are suppressed, thereby avoiding the loss of global information.

#### 2.3.4 Fine-grained score predictor

**Figure 6:**
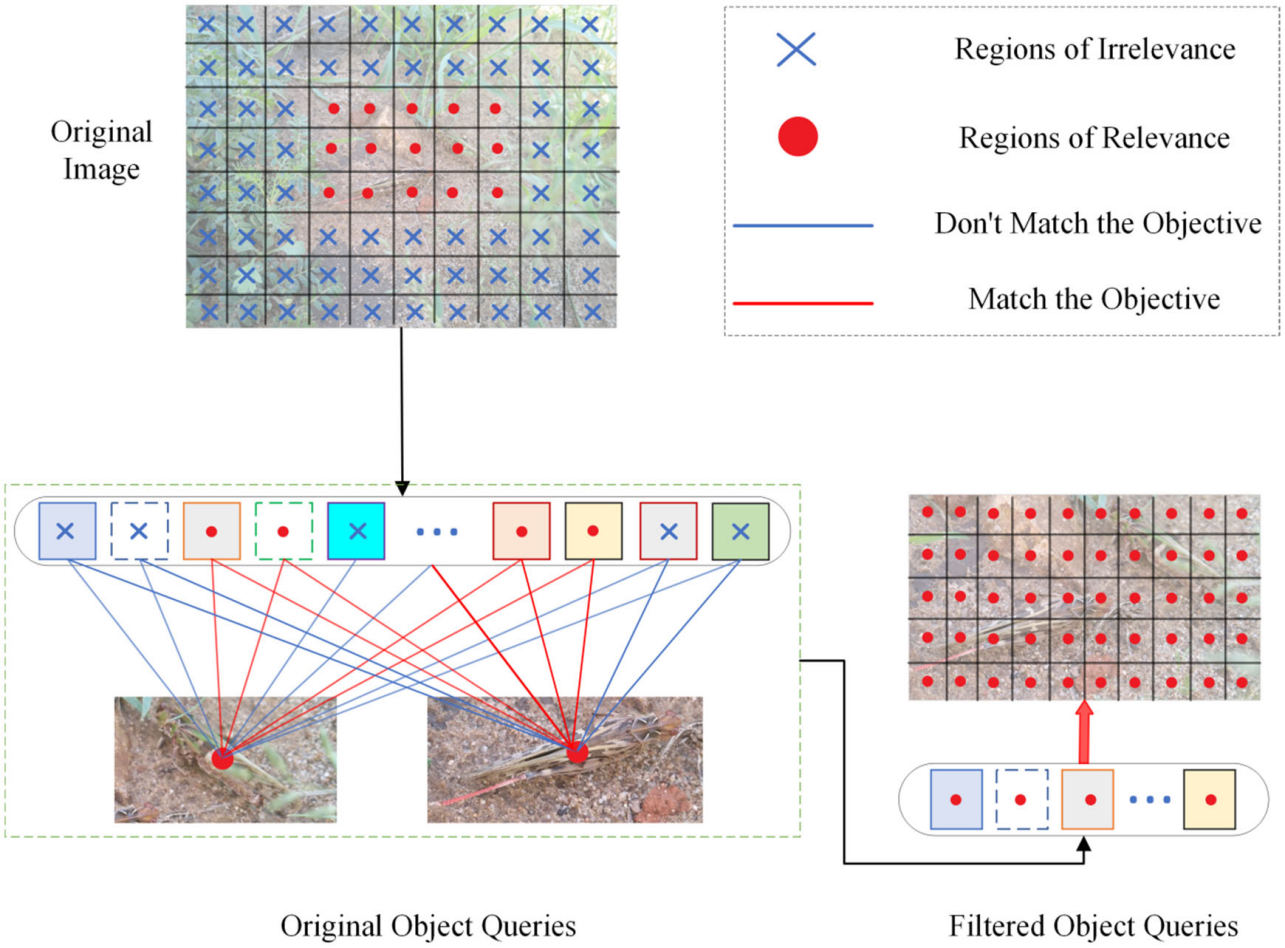
FGSP structure diagram.


After extracting multi-scale object features, we aimed to find a more detailed locust information extraction method to adapt to the locust’s changes in different environments. As shown in **Figure 6**, We proposed a fine-grained score prediction method based on the complex environmental background $P_{j}$ . This method further filters tokens extracted through multi-scale extraction, enabling the model to more accurately identify and focus on foreground objects. It helps in capturing the local features of objects more effectively, as shown in Equation 4:

(4) $$P_{j}=C_{j}\times S_{j}$$

Here, $C_{j}$ represents the probability of classes in the input image, and $S_{j}$ represents the probability of foreground scores in the input image.

Our model loss function defined as shown in Equation 5:


(5) ℒ=λmmℒ^atch+λddℒ^n+λmmMℒ^M+λffℒ^+λeeℒ^nc

where λmmℒ^atch is the loss based on Hungarian algorithm for bipartite matching, λddℒ^n is the loss of the denoising model, λmmMℒ^M is the loss of the MaskMlp, λffℒ^ is the loss of the foreground label selector, and λeeℒ^nc is the loss optimized through the output of the last encoder layer.

## 3 Experiments and results

### 3.1 Comparison with mainstream methods

To validate the versatility and effectiveness of the key-fg DETR model in the camouflage object detection task, we first conducted experiments on the publicly available COD10k dataset and then further tested the model’s robustness on the locust dataset. The experimental results and comparisons with other methods are listed in **Tables 3** and **4**. **Tables 3** and **4** show the performance comparisons between our proposed key-fg DETR, Deformable DETR (Zhu et al., 2020), DINO (Zhang et al., 2022), and Focus DETR (Zheng et al., 2023), Faster RCNN (Ren et al., 2015), RetinaNet (Lin et al., 2017), YOLOv5 (Jocher et al., 2022), and YOLOv8 (Reis et al., 2023). From the tables, it can be observed that key-fg DETR outperforms other detection models in camouflage object detection experiments, exhibiting higher recognition accuracy, the ability to learn meaningful feature distributions, and better adaptability to complex scene recognition tasks. Specifically, on the COD10k dataset, the constructed key-fg DETR model further enhanced the recognition ability for camouflage objects (see **Table 3**). Compared to other detection methods, our model shows significant improvements in metrics such as AP, AP_50_, and AP_75_. For instance, compared to Faster-RCNN, the AP value increased by 15.21%, AP_50_ by 15.73%, and Recall and F1-score improved by 7.90% and 10.27%, respectively. Compared to RetinaNet, the AP value increased by 19.61%, AP_50_ by 26.23%, and Recall and F1-score improved by 9.60% and 15.42%, respectively. Compared to EfficientDet, our model improves the AP value by 5.11%, AP_50_ by 3.53%, and Recall and F1-score improve by 13.60% and 12.05%, respectively. Additionally, Deformable DETR achieved an AP and AP_50_ of 23.07 and 60.59 at the 12th epoch. DINO achieved an AP and AP_50_ of 35.69 and 77.81 at the 24th epoch. Focus-DETR achieved an AP and AP_50_ of 35.81 and 78.06 at the 11th epoch. In comparison, our model achieved an AP and AP_50_ of 36.31 and 78.23 at the 12th epoch, with Recall and F1-score improving by 3.96% and 19.60% compared to Deformable DETR, 1.62% and 5.24% compared to DINO, and 0.50% and 3.39% compared to Focus-DETR.

**Table 3:** Comparison of the models on COD10k data.

Model	Epochs	AP	AP_50_	AP_75_	AP*_S_*	AP*_M_*	AP*_L_*	Recall	F1-score
Faster-RCNN	12	21.10	62.50	7.10	–	14.50	21.20	59.60	31.17
RetinaNet	12	16.70	52.00	4.00	–	9.10	16.80	57.90	25.92
YOLOv5	272	–	36.50	11.80	–	–	–	55.00	19.43
YOLOv8	200	–	40.10	14.90	–	–	–	56.10	23.55
EfficientDet	50	31.20	74.70	20.20	–	0.80	31.40	53.90	29.39
DeffusionDet	30	32.00	70.80	21.10	–	5.79	30.20	60.30	41.82
Deformable-DETR	10	23.07	60.59	13.18	–	3.59	23.22	63.70	21.84
DINO	24	35.69	77.81	30.50	–	2.99	35.94	64.00	41.31
Focus-DETR	11	35.81	78.06	27.04	–	4.60	36.09	64,30	38.07
key-fg DETR(Ours)	**12**	**36.31**	**78.23**	**29.90**	**-**	**2.50**	**36.59**	**67.50**	**41.44**

The meanings of the bold values provided are as follows:

Model: The name of the model used,

Epochs: The number of training epochs,

AP: Average Precision, measuring the overall detection performance of the model,

AP50: Average Precision at an IoU threshold of 0.5,

AP75: Average Precision at an IoU threshold of 0.75,

APS: Average Precision for small objects,

APM: Average Precision for medium objects,

APL: Average Precision for large objects,

Recall: Recall rate, representing the proportion of correctly detected objects,

F1-score: F1 score, a metric that considers both precision and recall.

**Table 4:** Comparison of the models on locust data.

Model	Epochs	AP	AP_50_	AP_75_	AP*_S_*	AP*_M_*	AP*_L_*	Recall	F1-score
Faster-RCNN	20	66.00	82.60	72.30	–	46.90	67.20	81.10	72.96
RetinaNet	12	58.00	80.50	66.40	–	24.00	59.70	78.20	66.51
YOLOv5	50	72.40	85.80	–	–	–	–	81.00	76.46
YOLOv8	50	73.50	85.30	–	–	–	–	80.60	76.90
EfficientDet	20	44.60	69.80	49.40	–	7.40	46.30	60.10	51.20
DeffusionDet	30	69.00	83.87	68.59	–	45.79	69.44	81.30	74,60
Deformable-DETR	31	62.12	86.84	69.34	–	36.79	63.15	78.90	69.60
DINO	18	73.26	86.33	74.95	–	62.00	74.10	81.60	77.19
Focus-DETR	14	73.31	86.45	75.21	–	60.65	74.12	81.70	77.25
DAB-Deformable DETR	21	72.82	87.02	74.64	–	58.34	73.81	80.80	76.58
key-fg DETR(Ours)	**16**	**75.07**	**87.57**	**76.66**	**-**	**60.96**	**75.84**	**81.80**	**78.19**

The meanings of the bold values provided are as follows:

Model: The name of the model used,

Epochs: The number of training epochs,

AP: Average Precision, measuring the overall detection performance of the model,

AP50: Average Precision at an IoU threshold of 0.5,

AP75: Average Precision at an IoU threshold of 0.75,

APS: Average Precision for small objects,

APM: Average Precision for medium objects,

APL: Average Precision for large objects,

Recall: Recall rate, representing the proportion of correctly detected objects,

F1-score: F1 score, a metric that considers both precision and recall.

In addition, in the experiments on the locust dataset, the recognition accuracy of this method has improved compared to other methods (**Table 4**). For instance, compared to Faster-RCNN, the AP value increased by 9.67%, AP_50_ by 4.97%, and Recall and F1-score improved by 0.70% and 5.23%, respectively. Compared to RetinaNet, the AP value increased by 29.29%, AP_50_ by 7.79%, and Recall and F1-score improved by 3.60% and 11.68%, respectively. Compared to EfficientDet, our model improves the AP value by 30.47%, AP_50_ by 25.77%, and Recall and F1-score improve by 21.70% and 26.99%, respectively. Additionally, Deformable DETR achieved an AP and AP_50_ of 62.12 and 86.84 at the 31st epoch. DINO achieved an AP and AP_50_ of 73.26 and 86.33 at the 18th epoch. Focus-DETR achieved an AP and AP_50_ of 73.31 and 86.45 at the 14th epoch. In comparison, our model achieved an AP and AP_50_ of 75.07 and 87.57 at the 16th epoch, with Recall and F1-score improving by 20.55% and 12.59% compared to Deformable DETR, 2.81% and 1.84% compared to DINO, and 2.43% and 0.94% compared to Focus-DETR. The average AP value of traditional detection models is 67.48, while that based on the DETR model is 70.38. The difference is primarily due to the distinct architectures of the two models. Traditional detection models rely on convolutional neural networks (CNNs) for feature extraction, which have certain limitations in detecting camouflaged objects in complex backgrounds. In contrast, the DETR model leverages attention mechanisms to better capture global information and complex contextual relationships, providing stronger feature extraction capabilities. Clearly, the DETR-based model has a greater advantage in locust detection.

### 3.2 Ablation experiment

To better demonstrate the advantages of the designed model, we conducted ablation experiments on four modules proposed for the key-fg DETR model, namely MaskMlp, Denoising Part, DropKey, and FGSP, to validate their effectiveness in camouflage object detection in complex scenarios. Our ablation study follows a progressive integration strategy, starting from the baseline model (Deformable DETR) and gradually adding each module to systematically validate its contribution and impact on the performance of camouflage object detection.

As shown in **Table 5**, we evaluated the model variants on both the COD10k and Locust datasets. The baseline model (EXP_A), corresponding to the original Deformable DETR, achieved an AP of 23.07 on COD10k (at epoch 10) and 62.12 on Locust (at epoch 32).

**Table 5:** Ablation experiment.

Experiment	Model	COD10k		Locust	
		Epochs	AP	Epochs	AP
EXP_A	Original	10	23.07	32	62.12
EXP_B	MaskMlp	11	23.88	46	64.89
EXP_C	MaskMlp+DN	11	33.71	34	73.26
EXP_D	MaskMlp+DN+Dropkey	8	34.95	45	74.44
**EXP_E**	MaskMlp+DN+Dropkey+FGSP	**12**	36.31	**16**	**75.07**

The meanings of the bold values provided are as follows:

Model: The name of the model used,

Epochs: The number of training epochs,

AP: Average Precision, measuring the overall detection performance of the model,

AP50: Average Precision at an IoU threshold of 0.5,

AP75: Average Precision at an IoU threshold of 0.75,

APS: Average Precision for small objects,

APM: Average Precision for medium objects,

APL: Average Precision for large objects,

Recall: Recall rate, representing the proportion of correctly detected objects,

F1-score: F1 score, a metric that considers both precision and recall.

In EXP_B, we incorporated the MaskMLP module. Unlike traditional attention mechanisms that uniformly weigh image regions, MaskMLP generates instance-aware attention masks through query-feature interactions, enhancing object localization and suppressing background noise. As a result, the AP increased to 23.88 (COD10k) and 64.89 (Locust), validating MaskMLP’s role in improving object-background separation. Further combining it with the denoising module (EXP_C) boosts the AP to 33.71 and 73.26, demonstrating its effectiveness in noise suppression and focus enhancement.

In EXP_C, we introduced the Denoising module to improve the stability of the matching process in Deformable DETR. This module mitigates the inconsistency of query-to-ground-truth matching and accelerates convergence by stabilizing offset learning. As a result, the AP significantly improved to 33.71 on COD10k and 73.26 on Locust, demonstrating the advantage of enhancing deformable attention with auxiliary denoising supervision.

In EXP_D, we added the DropKey regularization strategy. By introducing controlled sparsity in self-attention maps, DropKey selectively suppresses overconfident or noisy key positions without losing global context. This helps the model capture long-range dependencies better and improves robustness. The result was a further performance boost to 34.95 AP (COD10k) and 74.44 AP (Locust).

Finally, in EXP_E, we integrated the FGSP module. Unlike anchor-based query initialization in Conditional DETR (Meng et al., 2021) and DAB-DETR (Liu et al., 2022), FGSP introduces a spatial prior map adaptively generated from image content to guide queries toward potential foreground regions. This design is especially effective in camouflage scenarios where the foreground and background are highly similar. With FGSP, the AP values further increased to 36.31 (COD10k) and 75.07 (Locust). As shown in **Table 4** (EXP_D vs. EXP_E), FGSP improves spatial guidance under complex conditions, further enhancing the model’s focus and fine-grained feature perception.

**Figure 7:**
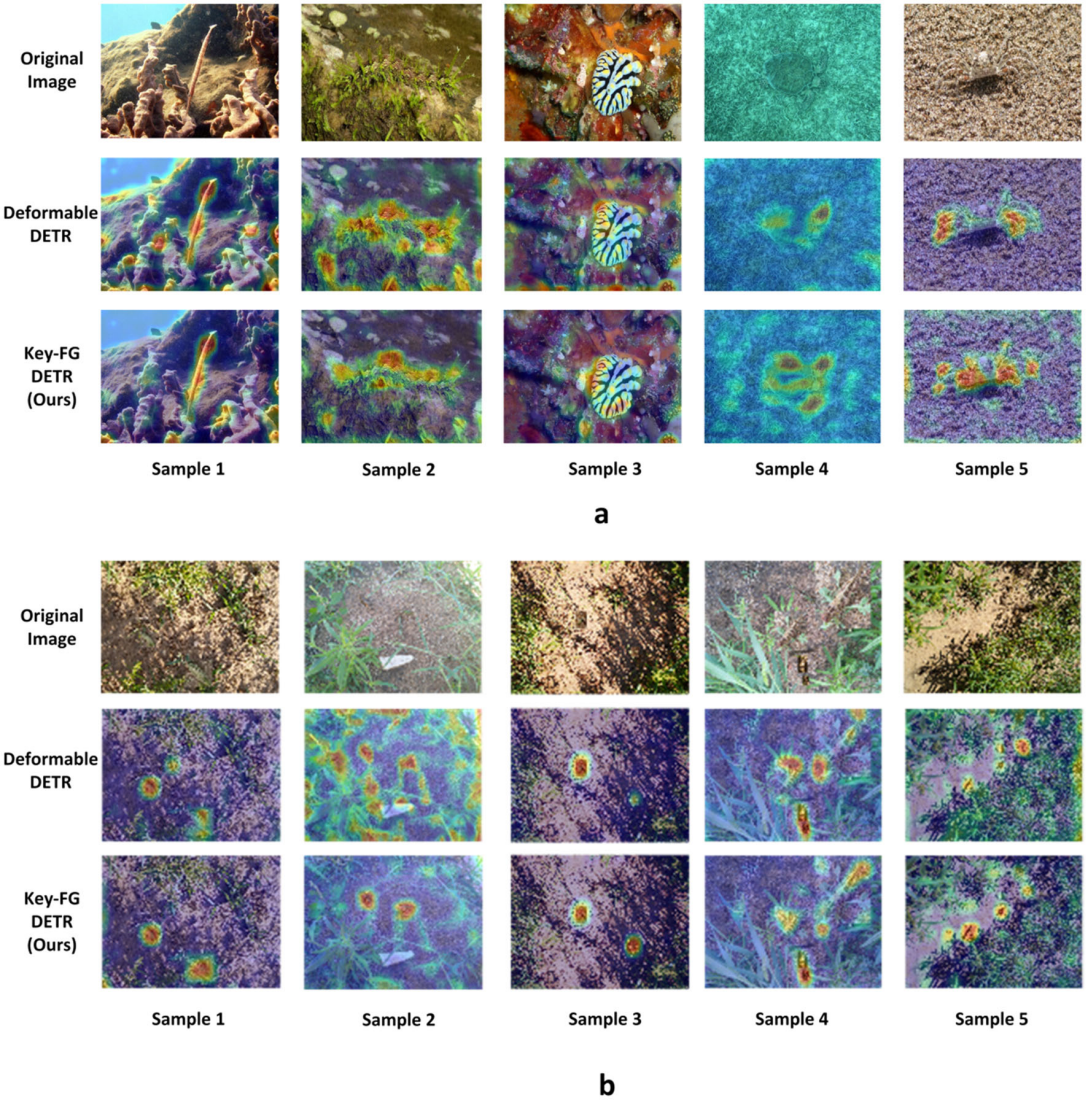
Comparison of heatmap visualization results **(a)** represent COD10k dataset and **(b)** represent locust dataset.

**Figure 8:**
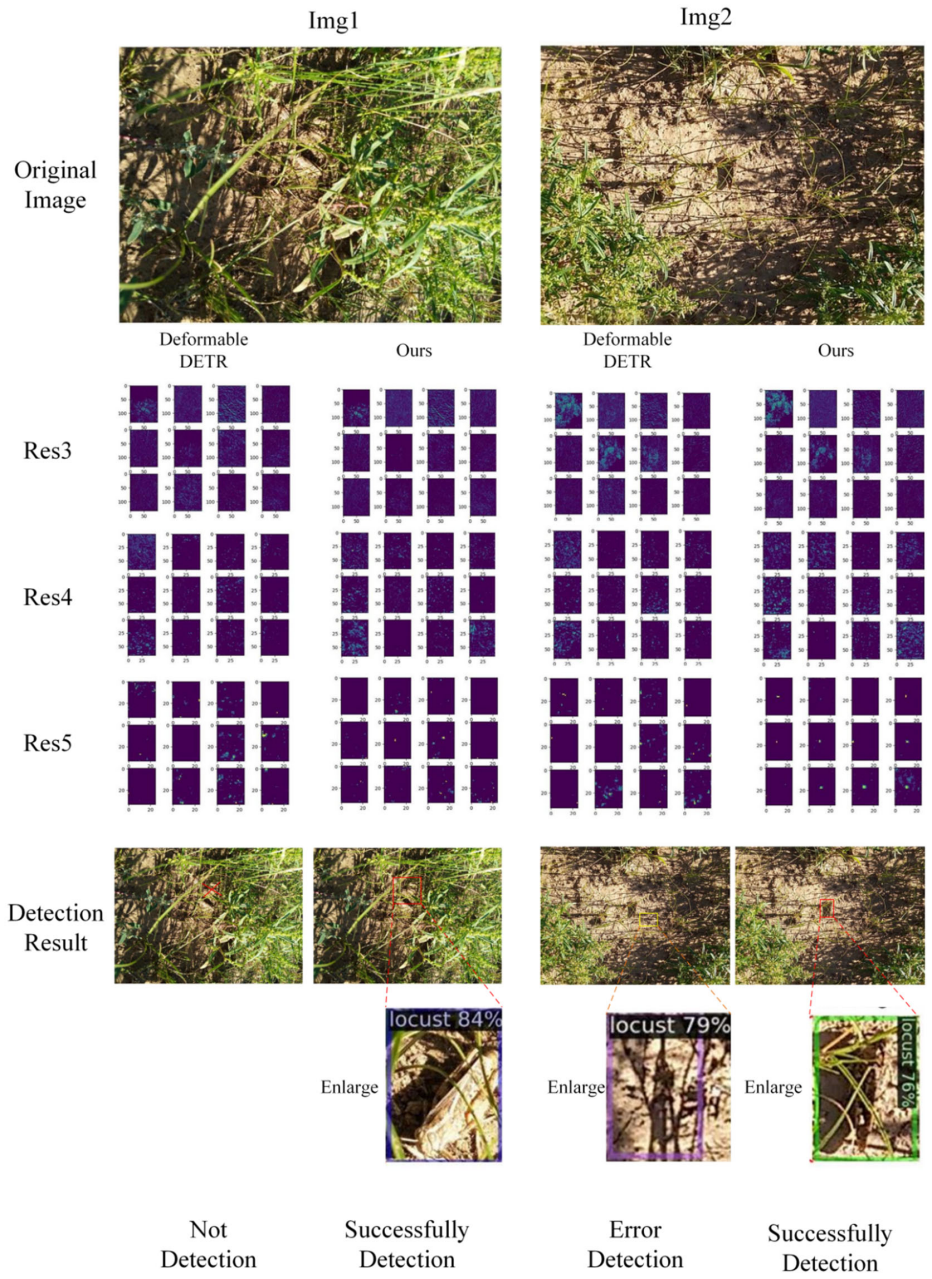
Visualization of the different feature layers.

**Figure 9:**
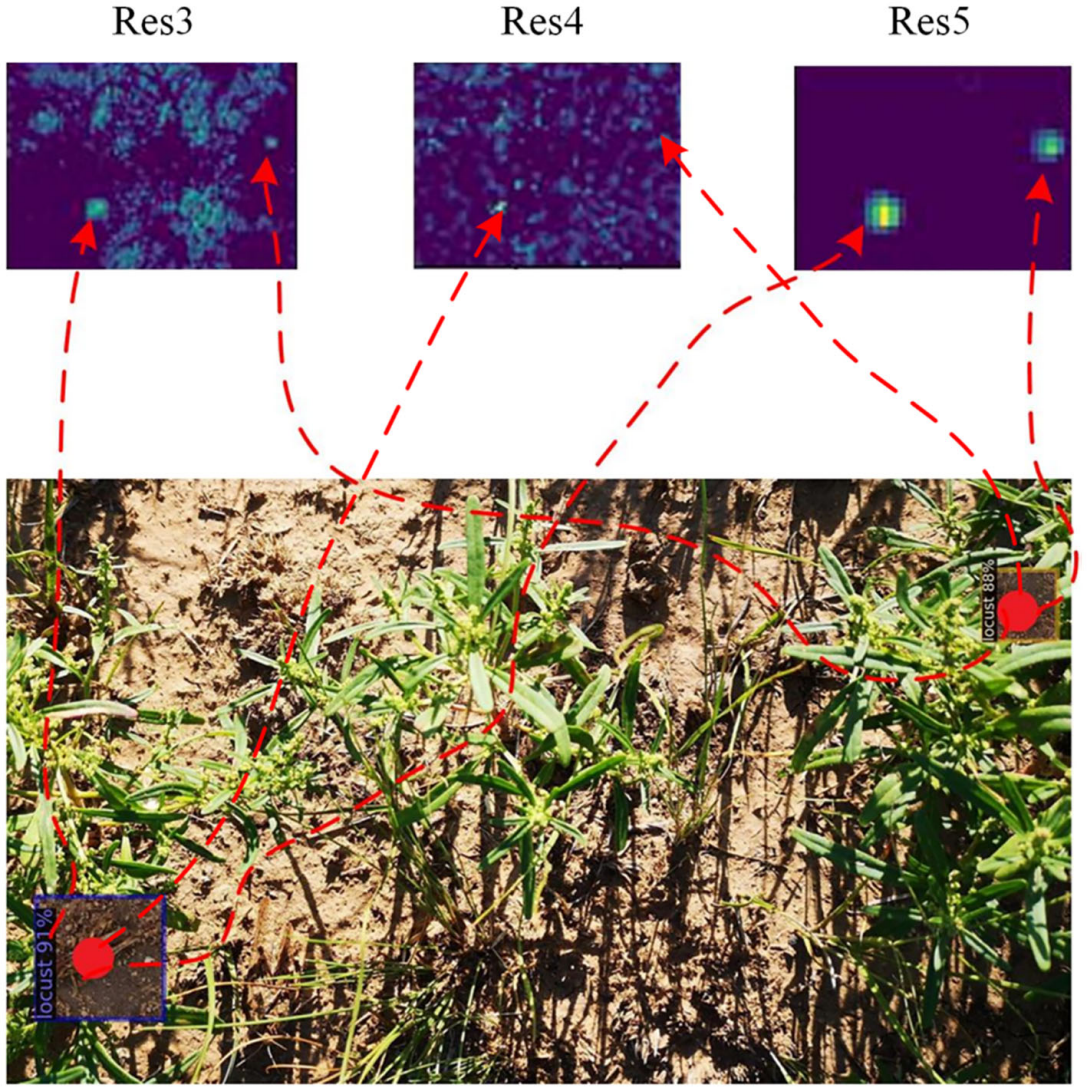
Local visualization of the different feature layers.

In summary, each proposed component contributes significantly to performance improvements. MaskMLP enhances instance-level discrimination; the denoising task accelerates learning stability; DropKey improves attention reliability; and FGSP refines spatial awareness and foreground focus. These modules are not simple plug-ins but carefully designed strategies tailored for camouflage object detection, as further supported by qualitative visualizations in **Figures 7**, **8**, **9**.

### 3.3 Visualization of detection results

To visually present the detection results of camouflage regions of interest, we conducted a visualization analysis of key-fg DETR (Ours) and Deformable DETR on the COD10k and locust datasets. The display results are organized into three modules: original image, Deformable DETR heatmap, and key-fg DETR heatmap.

**Figure 10:**
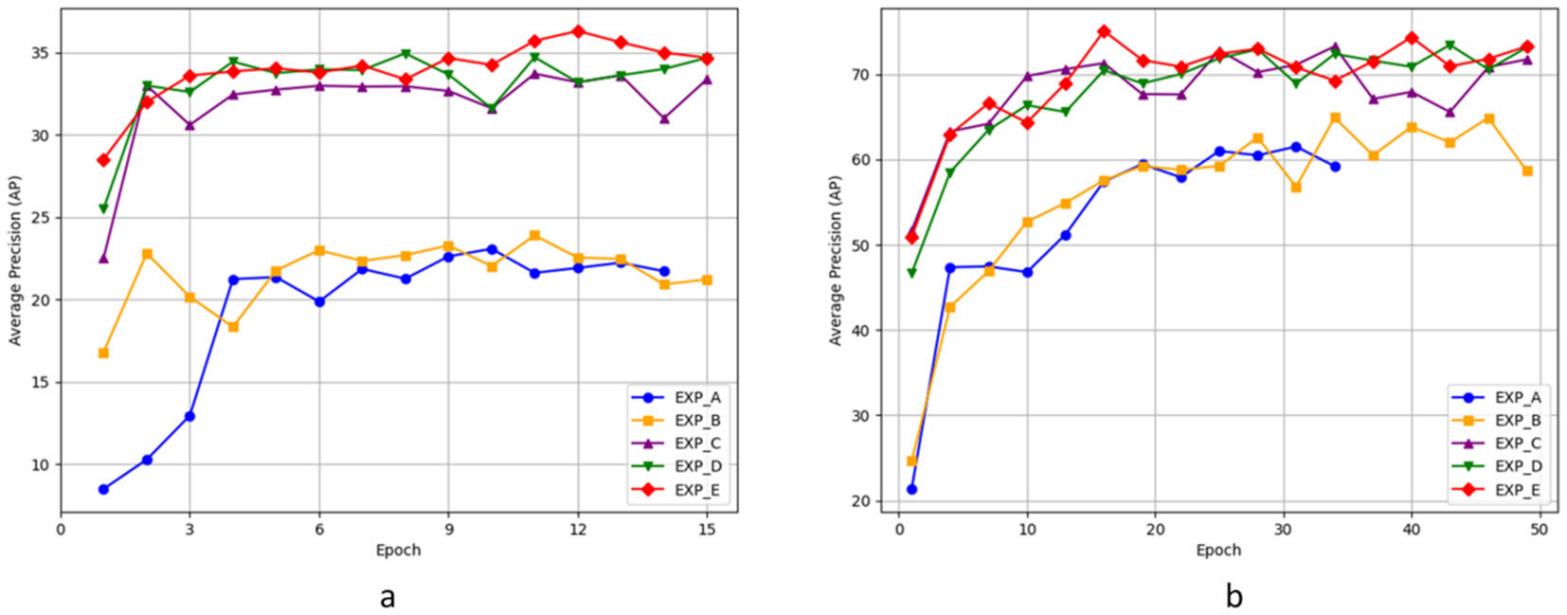
The AP plot of the ablation experiment.

As shown in **Figure 10**, we illustrated a comparative analysis of camouflage object detection performance between Deformable DETR and key-fg DETR on the COD10k and locust datasets. (a) shows the results on the COD10k dataset, and (b) shows those on the locust dataset. In each module, the first row shows the original image, the second row displays the Deformable DETR heatmap, and the third row shows the key-fg DETR (Ours) heatmap. From the figures, we can observe that, in the first two columns, Deformable DETR exhibits significant limitations in detecting camouflage objects. Its heatmap reveals that the object areas are scattered, with unfocused attention and substantial background interference, leading to misidentification of background areas that resemble the object. In contrast, key-fg DETR effectively concentrates on the object area, reducing background interference and significantly lowering false detection rates, demonstrating stronger object recognition capability. In the last three columns, Deformable DETR continues to struggle with object region recognition, showing blurred boundaries and difficulty distinguishing objects from the background. On the other hand, key-fg DETR achieves more precise object localization and boundary recognition, significantly improving detection accuracy and effectively avoiding missed detections.

Overall, key-fg DETR excels in handling complex backgrounds and camouflage object detection, achieving more accurate object identification and background elimination, which significantly enhances the overall performance of camouflage object detection.

To visually assess the detection accuracy, we showcased the detection results of Deformable DETR and the proposed key-fg DETR depicted in **Figure 8**. The first row of **Figure 8** displays the original locust images. The second to fourth rows of **Figure 8** display the visualized outputs of the third, fourth, and fifth layers of the backbone, respectively. The last row presents the detection results of the locust images. For Img1, Deformable DETR (column 1 of **Figure 8**) failed to detect locusts, whereas key-fg DETR (column 2 of **Figure 8**) successfully detected them. Regarding Img2, Deformable DETR (column 3 of **Figure 8**) mistakenly identified branches as locusts, while key-fg DETR (column 4 of **Figure 8**) accurately detected the locusts.

**Figure 9** displays the localized zoom-in visualization results of different layers in the key-fg DETR backbone network. From left to right are Res3, Res4, and Res5. Res3 corresponds to low-level features, typically associated with the shallower outputs in the network. It mainly extracts low-level features of the input image, such as edges, textures, and colors. From **Figure 9**, it can be observed that this layer first eliminated background areas with significant differences from the object, such as weeds. Res4 extracted features that were more advanced than Res3, including more complex shapes and structural information. By weakening the relationships between land and weeds in the background, Res4 could better capture the local details of locusts. Res5 extracted the highest-level features, usually corresponding to the global information and high-level semantic concepts of the input image. From **Figure 9**, it was evident that Res5 removed background interference and accurately located the locust information. The above results further demonstrated the advantages of the model in detection and localization of locust.

## 4 Conclusion

The accurate identification of camouflaged pest targets in real agricultural environments is a significant challenge, leading to substantial crop losses. This chapter uses the locust dataset as an example, as locusts are highly destructive pests that pose a serious threat to global food security. Therefore, accurate detection of camouflaged pests is crucial for pest control and sustainable agriculture. To address this issue, this chapter introduces a quantitative index for camouflaged object detection, the Camouflage Degree Index (CD), which evaluates the effectiveness of camouflage by calculating the feature differences between the target and the background. Based on the analysis of CD, the camouflage degree of locusts is found to be 1.05, surpassing the CD value of the classic camouflage object dataset COD10k.

To address this challenge, this paper proposes a Transformer-based detection method for camouflaged objects—key-fg DETR, which integrates techniques such as the FGSP module, MaskMlp, Transformer architecture, denoising mechanism, and DropKey. Through comparative experiments and ablation studies on both the locust dataset and the COD10k dataset, we validated the effectiveness of the model. Experimental results show that on the locust dataset, the AP value improves by 12.95 percentage points compared to Deformable DETR, and on the COD10k dataset, the AP value increases by 13.24 percentage points, demonstrating the strong capabilities of the model in both agricultural pest detection and camouflaged object detection. Additionally, the model shows significant advantages in common evaluation metrics, such as AP_50_, AP_75_, recall, and F1-score. Specifically, on the Locust dataset, recall increased by 6.15%, and the F1-score improved by 6.52%.

These results demonstrate that key-fg DETR can effectively detect camouflaged pests in challenging environments, significantly improving detection accuracy while reducing false positives and false negatives. Ablation studies further confirm the contributions of each module, proving that the FGSP module accurately guides the target region, the MaskMLP module generates instance-level masks, the denoising mechanism enhances training stability, and the DropKey strategy improves attention robustness.

This study not only provides strong algorithmic support for real-time detection of camouflaged pests but also contributes new insights to the development of precision agriculture technologies. The findings offer an effective tool for the early detection and control of agricultural pests such as locusts, which is crucial for crop protection and promoting sustainable agricultural development. Moreover, the proposed model is adaptable to complex environmental variations, demonstrating its broad potential in real-world applications and making a positive contribution to ecological protection and global food security.

## Data Availability

The original contributions presented in the study are included in the article/supplementary material. Further inquiries can be directed to the corresponding author.
